# An E-Learning Adaptation of an Evidence-Based Media Literacy Curriculum to Prevent Youth Substance Use in Community Groups: Development and Feasibility of REAL Media

**DOI:** 10.2196/12132

**Published:** 2019-05-09

**Authors:** Anne E Ray, Kathryn Greene, Michael L Hecht, Sarah C Barriage, Michelle Miller-Day, Shannon D Glenn, Smita C Banerjee

**Affiliations:** 1 Department of Health Behavior, Society, and Policy School of Public Health Rutgers, The State University of New Jersey Piscataway, NJ United States; 2 School of Communication and Information Rutgers, The State University of New Jersey New Brunswick, NJ United States; 3 REAL Prevention LLC Clifton, NJ United States; 4 College of Nursing Rady Faculty of Health Sciences University of Manitoba Winnipeg, MB Canada; 5 Department of Communication Studies School of Communication Chapman University Orange, CA United States; 6 Department of Psychiatry and Behavioral Sciences Memorial Sloan Kettering Cancer Center New York, NY United States

**Keywords:** substance use, prevention, media literacy, e-learning, adaptation

## Abstract

**Background:**

There is a need for evidence-based substance use prevention efforts that target high school-aged youth that are easy to implement and suitable for dissemination in school and community groups. The Youth Message Development (YMD) program is a brief, four-lesson, in-person curriculum that aims to prevent youth substance use through the development of youth media literacy. Specifically, YMD aims to increase understanding of advertising reach and costs, along with the techniques used to sell products; develop counterarguing and critical thinking skills in response to advertisements; and facilitate application of these skills to the development of youth-generated antisubstance messages. Although YMD has demonstrated evidence of success, it is limited by its delivery method and focus on alcohol and smoking.

**Objective:**

Study objectives were two-fold: (1) to adapt the YMD curriculum to a self-paced, interactive, electronic-learning (e-learning) format and expand its content to cover alcohol, combustible cigarettes, e-cigarettes, smokeless tobacco, marijuana, and prescription drugs, and (2) to test the feasibility of the adapted curriculum in partnership with a national youth organization.

**Methods:**

An iterative process was employed in partnership with the 4-H youth development organization and a technology developer and consisted of six phases: (1) focus groups to guide adaptation, (2) adaptation to an e-learning format renamed REAL media, (3) pilot-testing of the REAL media prototype to determine feasibility and acceptability, (4) program revisions, (5) usability testing of the revised prototype, and (6) final revisions. Focus groups and pilot and usability testing were conducted with 4-H youth club members and adult club leaders.

**Results:**

Focus group feedback guided the build of an e-learning prototype of REAL media, which consisted of five online levels and interactive content guided by a mix of narration and on-screen text. Results of a pilot test of the prototype were neutral to positive, and the program was refined based on end-user feedback. An independent usability test indicated that youth 4-H members felt favorably about navigating REAL media, and they reported high self-efficacy in applying skills learned in the program. Additional refinements to the program were made based on their feedback.

**Conclusions:**

The iterative build process involving the end user from the outset yielded an overall successful technology-driven adaptation of an evidence-based curriculum. This should increase the likelihood of effectively impacting behavioral outcomes as well as uptake within community organizations.

## Introduction

National survey results show a significant increase in prevalence rates of youth substance use during high school, which typically spans ages 14 to 18 in the United States [1,2]. For example, recent Monitoring the Future data [2] indicate that by 12th grade, 61% of youth have consumed alcohol, 45% have used marijuana, 36% tried vaping (including nicotine, marijuana, and/or just flavoring), 27% smoked cigarettes, and 11% have used smokeless tobacco. These figures represent a two- to three-fold increase from lifetime prevalence rates reported in eighth grade. This is of particular concern given the growing body of research that suggests there are detrimental effects on brain development among youth in this age range [3-5]. Research also shows that there is an inverse relationship between age of onset of substance use and risk for problematic use later in life [6].

Given that youth experimentation with substance use begins in early adolescence (or middle school), the majority of evidence-based substance use prevention efforts target youth of this age to prevent further escalation of use [7-9]. Yet, the transition to high school and the corresponding rise in prevalence rates nationwide suggest this may provide another meaningful opportunity for intervention. Given that fewer evidence-based options exist for high school-aged youth, there is a need for prevention efforts among this population to complement programming delivered earlier in adolescence [10,11]. Brief, theory-driven approaches that can be easily implemented with fidelity, require minimal resources, and are suitable for dissemination among community groups are important given the limitations of school- and community-based implementation [12].

Youth Message Development (YMD) is one example of an evidence-based program targeting youth in middle adolescence [13,14]. YMD is a brief, developmentally appropriate intervention for early high school-aged youth (ages 13-15 years) that aims to prevent adolescent substance use via increasing media literacy skills. A large body of evidence suggests that youth exposure to substance-related advertisements is associated with actual use [15]. YMD content (1) increases youth awareness of advertising reach and costs, (2) increases their knowledge of techniques advertisers use to sell products, (3) develops youth counterarguing and critical thinking skills in response to advertisements, and (4) includes an active learning component in which youth apply these skills and techniques to create and disseminate their own antisubstance messages [14]. This approach is developmentally appropriate because it responds to youth increases in executive function, independence, and rebelliousness that occur during middle adolescence. It also capitalizes on adolescents’ increasing media focus and social media connectedness [16]. Guided by the Theory of Active Involvement, YMD is thought to impact youth behavior via engaging youth in the curriculum leading to an increase in knowledge and skills, followed by a period of reflection on one’s own behavior and subsequent change in expectancies and normative beliefs related to substance use [17].

Initial research on YMD focused on smoking-specific ads, and results indicated a positive impact on beliefs about smoking as well as intentions to smoke among youth who received YMD relative to controls [18,19]. A follow-up study focused on alcohol ads and demonstrated positive effects on youth self-efficacy to apply curriculum skills [13]. Whereas many existing prevention curricula are time-intensive and are school- and family-based, YMD is unique in that it is designed to be brief (90 minutes or less) and delivered by community groups. For wide-scale implementation and dissemination, the timing is a benefit, whereas the need for an in-person facilitator necessitates resources such as time and availability, as well as training to increase fidelity. Additionally, as youth begin to experiment with other substances (eg, marijuana and other types of tobacco products) [1,2], it would be useful to expand the focus of programming to include content beyond alcohol and smoking.

To overcome these challenges of YMD, this paper focuses on adapting YMD from an in-person to an electronic-learning (e-learning) program, as well as broadening its focus to include other commonly used substances in adolescence. Much of human interaction and information seeking is migrating to electronic format, particularly among this age group, and substance use prevention efforts are no exception [20,21]. This raises issues about developing practices for electronic delivery, and also for those migrating from print or face-to-face delivery. These issues are particularly challenging when addressing the needs of populations like youth who are “digital natives” [16] and for whom interactivity has been identified as a key component of any effective substance use prevention intervention [22]. The objectives of this research were to (1) adapt an evidence-based, in-person media literacy-based substance use prevention curriculum for youth ages 13 to 15 years (YMD program) to a self-paced, interactive, e-learning format for implementation and dissemination in a youth organization, and (2) test the feasibility of this e-learning approach via both pilot and usability testing among the target audience.

## Methods

### Overview of the Adaptation Process

**Figure 1:**
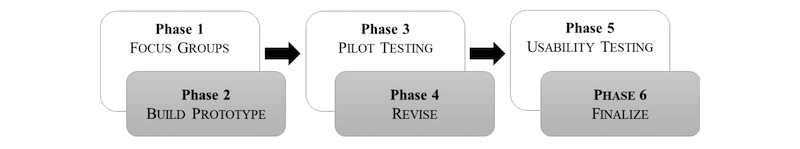
The six-phase adaptation process to convert Youth Message Development program to an e-learning format.

A six-phase process was employed to adapt the YMD curriculum from an in-person, paper-based curriculum to an e-learning program, and subsequently test the feasibility (see Figure 1): (1) focus groups were conducted to elicit feedback on existing content and suggestions for adaptation to e-learning, (2) an e-learning prototype was built in partnership with a technology firm, (3) the prototype was pilot-tested to establish feasibility, (4) modifications were made based on results from the pilot testing, (5) a usability test was conducted with a new set of users, and (6) the program was finalized based on usability findings. Phases 1, 3, and 5, which focused on gathering user feedback, were implemented with our target audience of 4-H youth members and adult club leaders, whereas phases 2, 4, and 6 were led by our technology partner. Thus, our adaptation process included a unique partnership model that went beyond adapting the evidence-based curricula with the help of a technology partner, and included the end user (in this case, 4-H members and leaders) from the outset. 4-H offers youth development programming via university-based cooperative extensions in communities throughout the United States and reaches nearly six million youth per year [23]. Their programming spans several areas including healthy living and incorporates a hands-on learning approach [24]. Accordingly, they were an optimal partner from both a content and dissemination standpoint. Recruitment and study procedures were approved by the Institutional Review Board of Rutgers University (New Brunswick, NJ).

### Participants and Recruitment

Participants for our user feedback phases (ie, focus groups, pilot testing, and usability testing) consisted of 4-H members (n=76) and leaders (n=16) recruited from clubs in New Jersey and Maryland (see Table 1 for demographics by phase). For each phase, club leaders were recruited via an email announcement from the state 4-H office. Interested leaders were then provided with a recruitment flyer to share with their teen members that described the purpose of the research activity, timing, and compensation. Flyers targeted high school-aged youth in grades 9 and 10. Parental consent and teen assent were obtained for all 4-H members, and informed consent was obtained from all 4-H leaders. Participants were provided with food during each user feedback phase, and members and leaders received US $30 and US $50 gift cards, respectively, as compensation for their participation.

**Table 1:** Participant demographics by phase.

Variable		Focus groups^a^ (n=27), n (%)	Pilot testing^a^ (n=43), n (%)	Usability testing^b^ (n=22), n (%)
**Participant type**				
	Members	19 (70)	38 (88)	19 (86)
	Leaders	8 (30)	5 (12)	3 (14)
**Gender**				
	Female	14 (52)	27 (63)	15 (68)
	Male	13 (48)	16 (37)	7 (32)
**Ethnicity**				
	Hispanic or Latino	6 (22)	3 (7)	0 (0)
	Not Hispanic or Latino	21 (78)	36 (84)	22 (100)
	Unknown	0 (0)	4 (9)	0 (0)
**Race**				
	American Indian/Alaska Native	0 (0)	1 (2)	0 (0)
	Asian	2 (7)	2 (5)	0 (0)
	Black or African American	1 (4)	7 (16)	3 (14)
	White	18 (67)	24 (56)	19 (86)
	More than one race	0 (0)	2 (5)	0 (0)
	Unknown	6 (22)	7 (16)	0 (0)

^a^Location: New Jersey.

^b^Location: Maryland.

### Procedures and Measures by Phase

#### Phase 1: Focus Groups

Four 2-hour focus groups were conducted with 4-H members and leaders to generate key ideas to guide the development of the e-learning version.

##### Procedures

Focus groups were led by the study principal investigators and cofacilitated by either the study project manager or a graduate research assistant. Focus group participants read copies of the in-person YMD curriculum, and subsequent discussions centered on improvements to curriculum content, including the use of acronyms and illustrative advertisements, framing of content, and providing ideas for transferring the content to an online platform (ie, use of voiceovers, program pacing, and various interactive features). Input was also solicited regarding the new program name. All focus group sessions were audio recorded and later transcribed. Detailed notes were taken for each session by the cofacilitator and circulated to the lead facilitator after each focus group to confirm accuracy.

#### Phase 2: REAL Media Development

The YMD curriculum was adapted to an e-learning prototype in a collaborative effort between the research team and technology developer.

##### Youth Message Development

The YMD curriculum consists of four lessons, is approximately 90 minutes in length, and is delivered in-person by a trained facilitator either all at once or in multiple sessions. Lessons focus on (1) media reach and strategies advertisers use to sell products, (2) claims in advertisements and counterarguments to those claims, (3) production techniques advertisers use to get attention (eg, setting, colors, font size), and (4) the application of content learned in lessons 1 to 3 to the development of a drug prevention message in the form of a poster [13,14].

##### Procedures

The development of the e-learning version of YMD, named REAL media based on focus group input and project team discussions, was an iterative process. Guided by the YMD curriculum, as well as feedback from the focus groups, the research team developed scripts for each lesson to guide the translation of content to e-learning format including on-screen text, narration, and interactive components. The Web development team offered its own expert feedback, and the entire team worked together to clarify the vision for REAL media. After each lesson was developed, the research team would provide feedback and the Web development team would make additional modifications. This process continued until the content was accurate and any observed technical glitches were resolved.

#### Phase 3: Pilot Testing

Five 2-hour pilot-testing sessions were conducted with 4-H leaders and members to assess the feasibility and acceptability of the REAL media prototype.

##### Procedures

Each participant was provided with a laptop computer with a wireless internet connection, mouse, and headset. Participants were asked to complete, at their own pace, as much of the REAL media program as possible during the 2-hour session. Participants were not asked to create an antisubstance use message—the final component of the REAL media program—due to time constraints, but they were provided with sample files to upload to test the program’s functionality. Participants also completed a brief survey to evaluate the performance of and their engagement in each level, what they liked and did not like, as well as demographic items. This was followed by a participant debrief led by the research team.

##### Measures

The 4-H members’ engagement at the end of each level was assessed with 12 items adapted from the Audience Engagement Scale [25] and Narrative Engagement Scale [26]. Participants were asked to indicate their agreement with each item on a 5-point scale from strongly disagree to strongly agree. Six items were from three subscales of the Audience Engagement Scale, including personal reflection (eg, “This level made me think a lot about my substance use [drugs, alcohol, tobacco]”), perceived novelty (eg, “This level was just like what we normally do in school”), and critical thinking (eg, “This level made me think about the truthfulness of ad claims”). The remaining six items were from three scales of the Narrative Engagement Scale, including interest (eg, “This level held my attention”), realism (eg, “The information in this level was very realistic”), and identification (eg, “The information in this level was relevant for me”). 4-H leaders responded to parallel items, which were adjusted to reflect their perception of how 4-H members would respond (eg, “They would think the information in this level is very realistic”). Open-ended feedback also was solicited. After each level was completed, participants were asked to respond to two prompts that captured (1) what they liked best about the level and (2) suggestions for improvement.

#### Phase 4: Revision to REAL Media

Changes were made to the prototype based on feedback from the pilot-testing sessions.

##### Procedures

The research team communicated the suggested changes to the programmer, who in turn made the edits before the usability test. The team verified the changes and performed internal testing before moving onto the next phase.

#### Phase 5: Usability Testing

After revisions to the prototype were made, an independent usability test was conducted with a sample of 4-H members and leaders with no prior knowledge of the REAL media adaptation process.

##### Procedures

Procedures for the usability test were similar to the pilot-testing procedures. Each participant was provided with a laptop computer with a wireless internet connection, mouse, and headset, and asked to complete levels 1 to 4 of the REAL media program during the 2-hour session. Participants were not asked to complete level 5 due to time constraints. Participants completed a brief survey to evaluate the usability after each level and the overall program.

##### Measures

Program usability of each level was assessed with 10 items adapted from the System Usability Scale (SUS) [27]. Sample SUS items were “I thought this level was easy to use” and “I found the various functions in this level were well integrated.” Coefficient alphas for SUS scores by level ranged from .83 to .92. Usability of the overall program was assessed with 19 items adapted from the Computer System Usability Questionnaire (CSUQ) [28]. Items fit into one of three subscales including system use (eg, “Overall, I am satisfied with how easy it is to use this program”), information quality, (eg, “The information provided for the program is easy to understand”), and interface quality (eg, “I like using the interface of this program”). Participants were asked to indicate their agreement with each item on a 5-point scale from strongly disagree to strongly agree. Mean scores were created for each subscale, and a total score was computed as the mean of all items. Coefficient alphas ranged from .89 to .98. Participants were also asked to list three positive and negative aspects of the overall program. Finally, self-efficacy to apply curriculum concepts was assessed among 4-H members only. This measure consisted of four items created for this study (eg, “I am confident that I can use these lessons to create my own counterarguments”).

#### Phase 6: Finalizing REAL Media

A list of changes to be made to REAL media before conducting a large-scale evaluation were identified.

##### Procedures

After the usability test, the research team analyzed the findings and documented a list of recommended changes to be made upon securing funding to finalize the program and evaluate its efficacy. This list also included any lingering issues identified in the pilot test that could not be accommodated before usability testing.

## Results

### Phase 1: Focus Groups

Focus group feedback was solicited on curriculum content and format with considerable time spent brainstorming suggestions for adapting the face-to-face content to a Web-based platform. Feedback was consistent across both youth and leaders, and it was organized by type (eg, content vs technology).

#### Content Feedback

Much of the content-specific discussion focused on the advertisements depicted in the curriculum, including their appropriateness and relevance to the audience. Youth participants were able to identify sample ads they liked as well as ads that did not resonate with their age group. One of the concepts in the curriculum focuses on ads that use sex appeal as a strategy to sell products. Youth participants acknowledged the importance of including this imagery because it was an accurate representation of what they were exposed to, but also noted the need to avoid showing “overly” sexual images for the more conservative members and leaders and/or younger club members. For example, showing a male celebrity in an underwear advertisement was deemed acceptable only if the image was cropped above the waist.

Another focus of the content-specific conversations related to the need for acronyms that resonated with the audience, and to adjust existing labels for key concepts accordingly. For example, the original acronym for the four strategies advertisers use to sell products was FUGE, which stood for fun with the group, unexpected/humor, glamor/sex appeal, and endorsement techniques, respectively. Youth participants suggested swapping the “G” to an “S” to produce FUSE, which would be easier to remember and could even be represented with an animated fuse. They also suggested relabeling the “S” from sex appeal to style, to avoid terminology that might be deemed inappropriate.

#### Technology-Based Feedback

Participants had many suggestions for how to adapt the curriculum content to a Web-based platform, including the use of narration to minimize on-screen text, interactive activities or games to maximize user engagement, positive feedback to encourage users when completing activities, the need for user flexibility in navigating the content, and age-appropriate communication. For example, they suggested avoiding a narrator with an adult voice, but also cautioned against someone who sounded too youthful or kid-like. Similarly, they wanted the narrator to offer positive feedback for correct answers that was simple (eg, “good job” or “way to go”) without being overly enthusiastic. Examples of suggested interactions were to allow the user to be able to manipulate images or ads to better highlight relevant lesson concepts and/or maximize contrasts and to offer a variety of interaction types. Participants also suggested that some users might want additional information on topics of interest and asked that additional content be made available to those seeking it. Labels were offered for program features that would avoid a school-like feel. For example, “lessons” could be “levels” and a “quiz” at the end of each lesson could be called a “challenge.” Finally, participants suggested potential names for the curriculum.

### Phase 2: REAL Media Development

Guided by the focus group results as well as the iterative feedback process described in the Methods, a five-level (ie, lesson) e-learning course was produced. From submission of the first script to completion, the build process took approximately 12 weeks. The main concepts from the face-to-face curriculum were retained and presented via a mix of on-screen text and narration, the latter of which was recorded by a younger, college-aged female. Although the face-to-face YMD curriculum consisted of four lessons, the first lesson was split into two separate lessons for REAL media to achieve a better balance of content and timing throughout. Each lesson was projected to take approximately 20 minutes to complete. In addition to the on-screen text, there were several interactions per level, including drag and drop, multiple choice, fill in the blank, sliders, and hover/reveal. Each level concluded with a brief “challenge” in which users were asked to apply the core concepts illustrated in the level. Finally, to offer interested users the option to see additional content, levels included “optional depth” segments in which users could learn more about the topics covered in that level. Additional resources were also included with each level. The inclusion of optional depth and resources allowed the user to “customize” or “personalize” their experience, a technique found to increase engagement and effectiveness [29].

Once built, the five levels were hosted on the technology company’s Learning Management System that was programmed so that users would be able to log in to their own personal account page and access the program. Each level was locked, meaning that participants had to proceed through the program sequentially by level; they did not gain access to the next level until the prior one was completed. To accommodate the less tech-savvy users, written instructions and a video were placed on the Learning Management System home screen to guide users through the log-in process.

### Phase 3: Pilot Testing

#### Level Engagement Self-Report

Ratings on indicators of realism, interest, and identification for the audience were neutral to positive overall as reported by both members and leaders (see Table 2). Average member ratings for realism across levels mostly ranged between agree (=4) and strongly agree (=5). Average member ratings for identification were between the neutral point (=3) and agree (=4), but closer to agree. Average member ratings for interest were around the neutral point (=3).

**Table 2:** Means and SDs for engagement scales by level for 4-H members and leaders. The n varies for each level because participants did not rate any levels they did not begin.

Scale		Level 1, mean (SD)		Level 2, mean (SD)		Level 3, mean (SD)		Level 4, mean (SD)		Level 5, mean (SD)	
		Member (n=38)	Leader (n=5)	Member (n=38)	Leader (n=5)	Member (n=36)	Leader (n=5)	Member (n=34)	Leader (n=5)	Member (n=15)	Leader (n=1)
**Audience Engagement Scale**											
	Personal reflection	3.33 (0.87)	3.70 (0.67)	2.99 (0.84)	3.50 (0.61)	3.35 (0.94)	4.10 (0.74)	3.18 (0.80)	3.80 (0.67)	3.33 (0.72)	3.50 (—)
	Novelty	3.89 (0.87)	3.70 (0.67)	4.07 (0.82)	4.20 (0.84)	3.43 (1.04)	4.10 (1.02)	3.91 (0.88)	3.90 (0.89)	3.60 (0.89)	2.00 (—)
	Critical thinking	4.18 (0.70)	4.40 (0.42)	3.86 (0.96)	4.40 (0.55)	4.31 (0.83)	4.50 (0.50)	3.96 (0.78)	4.00 (0.94)	3.77 (0.94)	4.00 (—)
	Total	3.80 (0.58)	3.93 (0.35)	3.64 (0.68)	4.03 (0.52)	3.69 (0.71)	4.23 (0.69)	3.68 (0.57)	3.90 (0.79)	3.57 (0.60)	3.17 (—)
**Narrative Engagement Scale**											
	Interest	3.01 (0.27)	2.80 (0.45)	2.92 (0.43)	3.10 (0.22)	2.99 (0.57)	3.20 (0.27)	2.88 (0.30)	3.00 (0.00)	3.13 (0.23)	3.50 (—)
	Realism	4.53 (0.65)	3.90 (0.89)	4.36 (0.56)	4.00 (0.94)	4.19 (0.68)	4.10 (0.65)	4.22 (0.68)	4.00 (0.71)	3.80 (0.32)	5.00 (—)
	Identification	3.76 (0.95)	4.00 (1.17)	3.76 (0.98)	4.20 (0.84)	3.50 (1.25)	4.10 (0.89)	3.94 (0.92)	3.90 (0.74)	3.63 (0.61)	2.00 (—)
	Total	3.77 (0.42)	3.59 (0.65)	3.68 (0.49)	3.77 (0.40)	3.56 (0.59)	3.80 (0.48)	3.68 (0.48)	3.61 (0.40)	3.51 (0.25)	3.50 (—)

Ratings on indicators of personal reflection on the impact of advertising and substance use, perceived novelty, and critical thinking about advertisements also were positive, with most averages for members close to agree (=4) for novelty and critical thinking. Member ratings for personal reflection were closer to the neutral point (=3), suggesting that not all members thought a lot about their own substance use after using the program.

Given the small sample of leaders, formal comparisons of their responses with those of members were not conducted. Nonetheless, means for leaders’ perceptions of personal reflection and critical thinking were higher than mean scores of members. Conversely, realism scores were higher for members than leaders.

#### Level Open-Ended Responses Feedback

Positive comments focused on content and technical aspects of the program. For example, specific to content, one user noted the examples were realistic and that made the program more relatable for teens. Comments related to technical aspects were focused on visuals, audio, interactive features, and overall program pace. Users reported the interactive features helped them to feel more engaged, the imagery helped keep their attention, the sound effects were positive, and the program was conducted at a nice pace.

Negative comments also focused on both content and technical aspects. Specific to content, participants noted words and concepts that were hard to understand. On the technical side, participants noted frustration with load time and other technical glitches. Other comments related to overall user experience focused on increasing flexibility, such as making it easier to go back to concepts they wanted to review. Although some participants noted they liked pacing, others noted it felt rushed. Other suggestions were to increase font size in certain places and to use more narration and less text.

### Phase 4: Revisions to REAL Media

**Figure 2:**
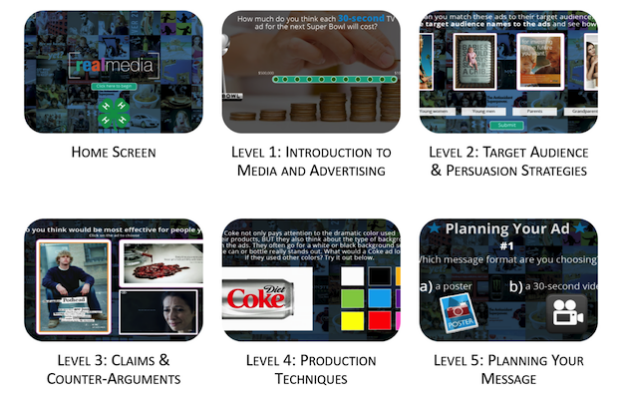
Images from the Revised REAL media prototype.

Based on feedback from the pilot test, edits were made following a procedure similar to the original development such that the research team reviewed the changes and offered feedback until minimal glitches were observed and the revised content was satisfactorily incorporated. Specific edits made included adding additional content to the log-in page to aid users in navigating the program (eg, explanations of program features), adding a feature so users could enlarge images, uploading higher quality versions of embedded video content, updating screens with new images, fixing minor content issues such as errors in grammar, fixing minor technical glitches (eg, voiceovers cutting off at the end of a screen), and adjusting contrast of text and/or background colors to enhance the visual experience. Figure 2 shows select screens from the revised prototype.

### Phase 5: Usability Testing

Means for all constructs assessed during the usability test are presented in Tables 3 and 4.

#### Usability

Mean SUS scores were favorable, with scores falling between agree (=4) and strongly agree (=5) for all levels (see Table 3). Scores for the CSUQ, which captured participants’ perceptions of the program, overall were also positive (see Table 4). Notably, both the SUS and CSUQ scores were higher among 4-H members than leaders, suggesting youth members had an easier time navigating the program overall.

#### Self-Efficacy

Ratings for confidence in using the information learned in REAL media to create counterarguments, use those arguments to convince others about media messages, use lessons to create advertisements, and change other people’s behavior via self-created advertisements were all high, with average scores falling between agree (=4) and strongly agree (=5) (see Table 4).

**Table 3:** Means and SDs for usability for 4-H members and leaders by level. The n varies for each level because participants did not rate any levels they did not begin.

Scale	Level 1		Level 2		Level 3		Level 4	
	Members (n=19)	Leaders (n=3)	Members (n=19)	Leaders (n=3)	Members (n=19)	Leaders (n=3)	Members (n=17)	Leaders (n=3)
SUS^a^, mean (SD)	4.24 (0.62)	3.93 (0.75)	4.28 (0.54)	3.93 (0.75)	4.10 (0.58)	3.07 (1.42)	4.28 (0.59)	3.73 (0.81)

^a^SUS: System Usability Scale.

**Table 4:** Overall program means and SDs for usability and self-efficacy for 4-H members and leaders.

Scale		Overall, mean (SD)	
		Members (n=19)	Leaders (n=3)
**CSUQ^a^**			
	System use	4.39 (0.82)	3.89 (0.96)
	Information quality	4.32 (0.84)	3.71 (0.62)
	Interface quality	4.40 (0.82)	3.83 (1.04)
	Total score	4.37 (0.80)	3.81 (0.85)
**Self-efficacy**			
	To create counterarguments	4.05 (1.13)	—
	To use counterarguments to convince others	4.26 (0.93)	—
	To create advertisements	4.21 (1.03)	—
	To change other people’s behavior	4.00 (1.20)	—

^a^CSUQ: Computer System Usability Questionnaire.

^b^Leaders did not complete this measure.

#### Open-Ended Responses Feedback

Similar to the pilot-testing feedback, comments from users on what they liked and did not like related to both technological aspects of the program and content. From a positive standpoint, participants liked the narration overall (eg, “it talks to you, keeps you involved”), visuals (eg, “pretty design,” “bright colors,” “cool pictures”), and the overall ease of use or navigation of the program (eg, “simple,” “easy to use”). Related to content, participants reported the program was informative, fun, and engaging, and liked the interactions.

Technological areas to improve included a more appealing sign-in page, as well as ongoing timing issues (eg, words went too fast on screen) and loading errors (eg, video would stop playing, some screens were slow). Some features were confusing and needed additional explanation (eg, the zoom button on images). Participants also noted the overall program could be shortened, particularly for a 2-hour session. From a content perspective, participants noted some sections were challenging (eg, the claims, evidence, and counterarguments), and they suggested refining the content presented to make concepts covered in this level easier to understand. Participants also requested more feedback from the program when they provided an answer (eg, they want to be told if they are right or wrong for open-ended responses).

### Phase 6: Finalizing REAL Media

Based on the feedback from both the usability and pilot, the research team identified further modifications for potential implementation in phase 2, the goal of which is to evaluate the impact of REAL media on user behavior prospectively and test the conceptual model guided by the Theory of Active Involvement as described previously.

From a technical perspective, a user-friendly log-in system is needed. Minor issues include edits to slide timing, transitions in content, and load time for each level.

From a content perspective, the youth requested more voiceovers, so there would be less on-screen text for users to read. In addition, we plan to streamline some of the repetitive content (particularly in level 3), which will be helpful for individuals who complete the program in one sitting. We will also offer more examples of challenging concepts, including claims, missing claims, and counterarguments (level 3). Finally, we plan to add a social media contest as an outlet for youth to share their antisubstance messages with their family and peers once they complete the e-learning curriculum.

## Discussion

Adolescent substance abuse remains a significant public health concern [1,2], and media literacy-based interventions appear to be a promising and novel approach to addressing this problem [13,17-19]. This study adapted a brief face-to-face media literacy alcohol and tobacco intervention for high school students (an underserved cohort in substance use prevention) for an online delivery system targeting multiple substances. Focus groups, a pilot study, and a separate usability study were conducted in the process of iteratively developing the curriculum. The resulting brief curriculum can be implemented in approximately 90 minutes (plus lesson 5 that occurs separately) or split into two to four separate lessons of approximately 20 to 25 minutes each, the latter of which is encouraged based on user feedback from pilot and usability tests. The findings highlight the potential for brief, focused active involvement interventions that can be applied to other substances as well as other public health concerns. In the discussion that follows, we highlight implications of this study and share lessons learned through employing a multiphase adaptation design that includes a partnership with a technology developer as well as the end user.

One of the main implications of these findings is the need for high levels of engagement with prevention materials. The potential for an online curriculum to meet these needs, and the challenges in doing so, are amply illustrated. This work identified both content and technical techniques for engaging the user. At the same time, the need for customization and personalization was also illustrated. For example, users want flexibility to navigate the program on their own terms, explore additional content if they choose, and receive personalized feedback on interactive elements including open-ended responses and some responses with feedback (eg, challenges).

One of the benefits of this innovative, brief, intervention component is that while potentially affecting substance use alone or in combination with a comprehensive intervention, it encourages the development of higher-order critical thinking skills valued in high school curricula under current teaching standards in most states. Favorable ratings on self-efficacy to use the skills REAL media aimed to build were notable and should only improve once additional edits are made particularly around the challenging concepts in level 3. This complementarity with curriculum standards should foster dissemination.

It is worth noting that the iterative process employed to adapt content to REAL media (ie, three data collections, three separate technology development phases) allowed the research team and technology developers to gain greater understandings of each other’s desires and limitations. Although extensive discussions occurred prior to initiation, it is our experience that differences in language, culture, and expectation require continued adjustments (and patience). Even though the technology company had considerable experience in training and implementation development, the media literacy approach was new to them and required considerable adjustments. Further, much discussion was required for both parties involved to understand respective goals. For example, our technology partner did not initially grasp our need for extensive program data capture to support self-report measures.

Overall, the curriculum demonstrated sound functionality and engagement through the iterative adaptation process. Although initial engagement ratings reported during the pilot test were not as high as we would have liked, improvements were made before usability testing. and the self-reported usability scores were very positive. We hope that additional changes scheduled to be made in response to usability feedback, as well as pilot feedback that was not able to be incorporated due to a short timeline, will further improve engagement. There may also be a ceiling effect for engagement in any intervention that has educational and prevention goals. It is an empirical question whether further improvements in engagement would yield increases in program outcomes.

We would also be remiss if we did not note the partnership model adopted throughout this research. Although the obvious partnership is between program developers (research team) and the technology developers, one cannot underestimate the importance of including the end user, in this case the 4-H organization, from the start. As opposed to the “build it, and they will come” model of intervention development, the research team adopted a partnership model that incorporates the end user from inception and has been applied successfully to other curricula adaptations [30]. As a result, REAL media is uniquely suited for dissemination through 4-H, which should facilitate the process of being taken to scale. Currently, 4-H clubs in nine states are using the curriculum in an efficacy trial to evaluate its impact on participants’ substance use.

Finally, there were a few limitations of note. Given the pilot nature of the study, we did not assess teen participants’ expectancies related to and actual use of substances. It is possible youth substance use tendencies and beliefs could influence their perceptions of the intervention. Accordingly, future feasibility studies on this topic should consider including these measures. In addition, participants were not selected at random. Thus, it is possible the individuals who self-selected into the study are not representative of 4-H participants as a whole. The sample was also small in size and predominately white. All these factors together potentially limit the generalizability of the results. The small sample size also limited our ability to look at differences in results between participating clubs.

In conclusion, this paper describes an iterative development process for adapting evidence-based, face-to-face, manualized prevention programming to an online format whereby the end user is involved from the outset. Both challenges and triumphs were experienced throughout the process, and efforts were generally successful overall. We believe that entering the field with a more fully developed curriculum increases the chances of effectively impacting behavioral outcomes as well as the likelihood of uptake.

## Abbreviations

CSUQ: Computer System Usability Questionnaire
e-learning: electronic learning
NCI: National Cancer Institute
NIDA: National Institute on Drug Abuse
NIH: National Institutes of Health
SUS: System Usability Scale
YMD: Youth Message Development
